# Cross-sectional validity and specificity of comprehensive measurement in lymphedema and lipedema of the lower extremity: a comparison of five outcome instruments

**DOI:** 10.1186/s12955-020-01488-9

**Published:** 2020-07-22

**Authors:** Felix Angst, Susanne Lehmann, André Aeschlimann, Peter S. Sandòr, Stephan Wagner

**Affiliations:** 1 Research department, Rehabilitation clinic (“RehaClinic”), Quellenstrasse 34, 5330 Bad Zurzach, Switzerland; 2 Department of angiology, Rehabilitation clinic (“RehaClinic”), Bad Zurzach, Switzerland

**Keywords:** Lymphedema, Lipedema, Validity, Construct, Convergent, Divergent, Discriminant

## Abstract

**Background:**

Literature on the validity of outcome measurement in lymphedema and lipedema is very sparse. This study aimed to examine the convergent, divergent and discriminant validity of a set of 5 instruments in both conditions.

**Methods:**

Cross-sectional outcome was measured by the generic Short Form 36 (SF-36), the lymphedema-specific Freiburg Quality of Life Assessment for lymphatic disorders, Short Version (FLQA-lk), the knee-specific Knee Outcome Survey Activities of Daily Living Scale (KOS-ADL), the Symptom Checklist-90-revised (SCL-90R), and the Six-Minute Walk Test (6 MWT). Construct convergent/divergent validity was quantified by bivariate correlations and multivariate factor analysis, and discriminant validity by standardized mean differences (SMDs).

**Results:**

Health was consistently better in lymphedema (*n* = 107) than in lipedema (*n* = 96). The highest construct convergence was found for physical health between the SF-36 and KOS-ADL (bivariate correlations up to 0.78, factor loads up to 0.85, explained variance up to 56.8%). The second most important factor was mental health (bivariate correlations up to 0.79, factor loads up to 0.86, explained variance up to 13.3%). Discriminant validity was greatest for the FLQA-lk Physical complaints (adjusted SMD = 0.93) followed by the SF-36 Bodily pain (adjusted SMD = 0.83), KOS-ADL Function (adjusted SMD = 0.47) and SF-36 Vitality (adjusted SMD = 0.39).

**Conclusions:**

All five instruments have specific strengths and can be implemented according to the scope and aim of the outcome examination. A minimum measurement set should comprise: the SF-36 Bodily pain, SF-36 Vitality, FLQA-lk Physical complaints, FLQA-lk Social life, FLQA-lk Emotional well-being, FLQA-lk Health state, KOS-ADL Symptoms, KOS-ADL Function, and the SCL-90R Interpersonal sensitivity.

## Introduction

Lymphedema and lipedema of the leg are burdensome chronic diseases, for which no curative treatment has yet been found [1–7]. Primary lymphedema is characterized by intrauterine malformation or genetic deformity with impaired lymphatic transport [1, 2]. Secondary lymphedema is caused by an ineffective lymphatic flow, mostly frequently the result of traumatic or iatrogenic lymphatic vessel interruption [1, 2]. As a consequence of reduced lymphatic transport interstitial fluid accumulates, with chronic swelling of the drainage region involved. For the diagnosis of lymphedema the patient’s case history and physical status are usually sufficient and specific enough [1–3]. There are no specific diagnostic tests for lymphedema.

Lipedema is characterized by the abnormal, disproportional deposition of subcutaneous fat in the extremities, leading to a disproportionate enlargement of the legs, and, in some cases, also the arms [4, 5]. Lipedema is always linked with daily pain, i.e. allodynia, ranging from disturbing heavy legs, pain on contact to pain that is permanent and disabling [4–7]. Lipedemia almost exclusively affects women and is probably due to hormonal stimulation and /or a genetic predisposition. It is not necessarily linked to obesity, but it may be induced and further aggravated by weight gain. Diagnosis is based on case history and clinical signs [5–7]. As for lymphedema, no technical or objective tests have yet been developed to confirm the diagnosis of lipedema. A differential diagnosis can therefore be challenging with the potential for misdiagnosis as lymphedema, obesity, or rheumatic diseases, such as fibromyalgia [4, 7].

Outcome data regarding the health and quality of life of patients with lymph- or lipedema of the lower extremities are scarce; some are limited to special syndromes [8–10]. In 2015, for example, we published a cross-sectional outcome report on primary and secondary lymphedema of the lower extremity [11]. That initial study measured health and quality of life, including social functioning and specific psychosocial and mental health dimensions, using a combination of comprehensive and condition-specific scales. We found that the health of primary lymphedema patients was unaffected when compared to specific population norms, whereas secondary lymphedema patients reported limited physical function and physical and emotional role performance compared to the norm. Our earlier research did not examine lipedema. A recent study presented cross-sectional data collected with the Short Form 36 (SF-36) in *n* = 18 patients with lymphedema together with cholestasis, a very rare syndrome [9]. The SF-36 findings of that study were similar to our own. A recent validation study by Van den Pas et al. of the Lymphedema Quality-of-Life Questionnaire (LYMQOL) showed only correlations and presented no descriptive data from the SF-36 [8].

A comparative understanding of the psychometric properties of different instruments’ scales is essential in order to further the aim of elaborated outcome measurement in any health condition [12]. In this context, validity plays the major role [12, 13]. To the best of our knowledge, for lipedema of the lower extremities no detailed data has yet been published on the measurement properties and, in particular, on the validity of comprehensive and specific outcome assessments. In lymphedema, the report by Van den Pas et al. 2016 mentioned earlier is the only comparable study providing an analysis of validity [8].

Our analysis aimed 1) to examine and to compare the cross-sectional validity of the measurement scales of different outcome instruments covering specific constructs of health dimensions (especially convergent construct validity) and 2) to determine their ability to specify and to differentiate between lymphedema and lipedema (discriminant construct validity).

## Methods

### Patients and data sampling

Patients were consecutively referred by their family physician, internist or angiologist to the angiology department of the Rehabilitation Clinic “RehaClinic”, Bad Zurzach, Switzerland for outpatient consultation or inpatient treatment. The examination of outpatients aimed to establish a plan for the future management of their condition by their family doctors and relevant therapists outside our clinic. Inpatient treatment consisted in intensive complex decongestive lymphatic therapy and comprehensive rehabilitation, mainly through aquatic and land-based physiotherapy. The Swiss health insurance companies reimbursed inpatient rehabilitation, on condition that patients were still suffering from symptoms needing further treatment, despite having received correct outpatient physiotherapy and compression therapy. The study was approved by the ethics committee of Aarau, Canton Aargau, Switzerland (EK AG 2008/026) and written informed consent was obtained from all study participants.

The inclusion criteria were as follows: age of 18 years or older, and a confirmed diagnosis of 1) lymphedema of the leg stage II–III, or 2) lipedema stage I–III, or 3) combined lip−/lymphedema, denoting advanced lipedema of the lower extremity (with secondary lymphedema characterizing the course) in accordance with the guidelines for the two syndromes [1, 5]. All diagnoses were made or confirmed by the head of the angiology department (SW). A diagnosis of secondary lower limb lymphedema required that the patient’s case history include one of the following explanatory causal events: trauma, surgery, neoplasm or its subsequent treatment [14, 15].

The exclusion criteria were the following: 1) Edema combined with a predominantly non-lymphatic or non-lipedema component, especially edema caused by venous insufficiency (>stage C2 according to the CEAP classification), cardiac or renal failure, or liver insufficiency [16]. 2) A body mass index (BMI) > 50.0 reflecting severe obesity, which has a major impact on the levels of health dimensions in contrast to the lipedema alone*.* 3) Mixed edema of unknown origin, and/or classification as lymphedema or lipedema was impossible. 4) Assessment impossible due to the patient’s insufficient knowledge of the German language, insufficient psycho-intellectual abilities, or severe somatic illness.

### Measures

Sociodemographic and disease-relevant data were recorded using a standardized questionnaire that has proved its worth in several of our previous studies [17]. All necessary medical records were obtained to enable confirmation of the diagnosis and evaluation of the inclusion and exclusion criteria and the number of comorbid conditions.

The following five instruments were used. The Short Form 36 (SF-36) is the questionnaire most widely used for the self-assessment of generic health and quality of life [18–20]. We used the revised version SF36-version 2 [19, 20]. The instrument’s 36 items build eight dimensions, namely, physical functioning, role physical, bodily pain, general health, vitality, social functioning, role emotional and mental health. A complex linear combination of the eight scales (each of which represents a content and construct dimension) forms two component summaries (physical and mental). The SF-36 is used world-wide, which facilitates good comparability among various health conditions [18].

The Freiburg Quality of Life Assessment for lymphatic disorders, Short Version (FLQA-lk) is comprised of 33 items composing six scales: physical symptoms, daily and professional life, social life, mental health, therapy of the lymphatic disorders and satisfaction [21]. The sum of the 33 items gives the total score. The FLQA-lk is, to our knowledge, the only validated disease-specific instrument for lymphatic disorders in the German language [11].

The Knee Outcome Survey Activities of Daily Living Scale (KOS-ADL) measures symptoms and quantifies the level of impairment due to knee pathologies (pain, swelling, stiffness, etc.) and the resulting functional restrictions affecting activities of daily living (stair-climbing, kneeling, etc.) [22–24]. The questionnaire is short (14 items) and has good psychometric properties [22]. The KOS-ADL was chosen because swelling of the lower extremity may lead to impaired knee function and symptoms. The subscale symptoms (items) and functions are summed up to give the total score. A numeric rating scale (0–100) quantifies the change of function in activities of daily living compared to the function pre-edema (0 = no function, 100 = function as before).

From the Symptom CheckList-90- Revised (SCL-90R), two further scales, measuring interpersonal sensitivity (nine items) and obsessive/compulsive (10 items), were selected. The SCL-90R is one of the best established tools for assessing psychiatric syndromes [25, 26]. Both syndromes mentioned may affect young women with lipedema or lymphedema, chronic conditions having a potential impact on body image and demanding strict therapy adherence. An altered body image due to swollen legs may affect interpersonal sensitivity and the demands of strict therapy adherence may lead to obsessive/compulsive signs. The above two scales measure constructs not covered by the mental health dimensions of the most commonly used instruments.

Finally, we applied the Six-minute Walking Test (6 MWT), one of the most frequently used and responsive functional performance tests [27–29]. All the instruments were implemented in their validated German versions.

### Analysis

The cross-sectional assessment of outpatients took place on the day of the first consultation and that of inpatients on the day of admission for therapy (before therapy). The instruments’ missing rules had to be fulfilled in order to determine valid scales for the analysis (program criteria). Thus, for each scale of the SF-36 patients had to complete more than 50% of the items [19, 20]. Since no missing rules were defined in the original descriptions and manuals of the other questionnaires used, the requirement was set at more than 66.7% (two thirds) completed items. This was the rule as originally applied in a similar study assessing outcome after total shoulder arthroplasty [30].

All scores were scaled from 0 = worst health, maximum symptoms/disability to 100 = best health, no symptoms, full function, as originally described for the SF-36. The one exception was the 6 MWT, where the walking distance was quantified in meters (m). Condition-stratified descriptive data included floor and ceiling effects (percentages) to quantify an instrument’s ability to depict the whole spectrum of the disease and to specify symptom or impairment levels.

In validity testing, the terms “content”, “criterion” and “construct” validity describe different focuses of testing and show overlapping aims and contents, which add and correlate together to provide an overall picture of measurement precision [12, 13, 31]. Content validity and reliability have been tested in the manuals and first presentations of the original questionnaires and their translations [18–29]. The SF-36, which has been used for more than three decades in thousands of studies and settings, serves as the “gold” standard for the examination of concurrent criterion validity, for example, to quantify the convergent validity of the complex construct of pain [19, 20, 31]. The content, criterion and construct validity of the SF-36 Mental health for the measurement of depression has recently been exhaustively demonstrated [32].

Correlation analysis (parametric product moment according to Pearson) and factor analysis were used to examine the construct convergence and divergence (or discriminant validity) of the scales [12, 13, 31]. The two resulting 24 × 24 half matrices of bivariate correlations for lymphedema and lipedema, i.e. 576 single correlation coefficients, are difficult to summarize and are shown in full in two separate appendices.

Factor analysis is a multivariate correlation analysis designed to reduce the number of dimensions and to specify common constructs [31]. The factor loads of different instruments’ scales quantify the convergent and divergent/discriminant validity of complex constructs, for example pain as a syndromic dimension [13]. Principal component factor analysis with varimax rotation and parallel analysis to determine the number of valid factors was used to provide the explorative characteristics for this purpose [31]. This technique determines common vectors that summarize the direction of several dimensions/scales. The orthogonal projection (cosines) of each scale on each vector (=factor), the “factor load”, reflects the construct convergence of the scale to the common dimension of the factor. Low correlations and factor loads reflect divergent construct validity [12, 13]. All the instruments’ summary and total scores were excluded from the factor analysis, because their constructs are already comprised in the single scores. The 6 MWT was also excluded, because walking distance data were available only for the inpatients (*n* = 89 lymphedema, *n* = 64 lipedema). Inclusion of those data would have restricted the analysis to the inpatients. Missing values were replaced by mean values of the subjects with completed scales. We used Velicer’s minimum average partial (MAP) test and parallel analysis as criteria to determine the number of factors to be retained [31]. Both criteria are upgraded methods of the somewhat outdated “Eigenvalue> 1.0″ criterion [31].

The bivariate comparison of the scores for lipedema and lymphedema was performed using standardized mean differences (SMDs), in order to quantify the instruments’ ability to specify and to differentiate between the two conditions (discriminant construct validity or “known groups” validity – a component of construct validity) [12, 13, 31]. SMDs are well-known measures of effect differences between verum (active study drug) and placebo and widely used in randomized controlled trials. In this study, SMDs were used as the standardized differences between two scores (lymphedema and lipedema), in line with the original description of their application [33].

Multivariate, adjusted SMDs were calculated by multivariate regression analysis, modeling the score difference (between lymphedema and lipedema) as dependent variable by the potential confounders age, education level, number of comorbidities, and in−/outpatient status as independent variables. Score differences that are independent from those co-variates can thus be obtained. Further substantial confounders are sex and BMI (being overweight). These were not included in the regression modeling, because they are defining characteristics of lipedema and related to its diagnosis.

In determining the sample size, the level of the difference in outcome between lymphedema and lipedema was an important consideration. In order to reach statistical significance for an SMD = 0.30, the sample size for each condition should be *n* ≥ 87 (minimal degrees of freedom = 87 + 87–2 = 172) [34]. This constitutes the lower limit (0.30) of the range of 0.30–0.50 currently considered to indicate minimum clinically important differences (MCIDs) [34]. In other words, above that level, differences become subjectively perceptible on the group level. Our sample sizes met this requirement. A doubling of the sampling effort and costs to increase the total sample size to 2*2*87 = 348 would narrow the upper and lower limits of the 95% confidence interval by only 0.075 each [33, 34]. Furthermore, in order to be sufficiently determined, every factor analysis should comprise at least 5 cases per variable, i.e. in our analysis, 5*19(scales) = 95 patients per diagnostic group with complete data in all scales [31].

## Results

### Patients

Table 1 presents the basic data of *n* = 107 patients with lymphedema and *n* = 96 with lipedema. Compared to lipedema, the lymphedema group was, on average, 9.7 years older, slimmer (BMI 6.7 lower) and also comprised men (29%). In−/outpatient status, educational level and number of comorbidities were similarly distributed.

**Table 1:** Socio-demographic and disease-relevant data (*n* = 175)

Characteristic	Lymphedema (*n* = 107)	Lipedema (*n* = 96)	p
Proportion of sample (%)	52.7	47.3	–
Outpatients (%)	54.2	56.3	0.532
Female (%)	71.0	100.0	< 0.001
Living alone (%)	25.0	16.1	0.392
Education (%)			0.310
Basic school (8–9 years)	6.6	11.0	
Vocational training	47.2	53.8	
College/high school/university	46.2	35.2	
Comorbidities (%)			0.577
none	4.8	8.5	
1	19.2	16.0	
2	21.2	19.1	
3	21.2	26.6	
4	14.4	12.8	
≥ 5	19.2	17.0	
BMI (kg/m^2^): mean (sd)	28.1 (8.7)	34.8 (8.6)	< 0.001
Age (years): mean (sd)	56.4 (14.8)	46.7 (13.7)	< 0.001

Legend: *BMI* Body mass index, *sd* Standard deviation, p = type I error (2-tailed, Chi-square or t-test for independent samples)

### Descriptive data

The score data on all instruments are shown in Table 2. There were few or no floor effects (maximum 6% on the SF-36 Bodily pain scale in lipedema). In lymphedema, ceiling effects were moderate (10–30%) on the SF-36 Role physical and Bodily pain scales, the FLQA-lk Social life and Treatment scales, the KOS-ADL Function and both SCL-90R scales; but they were high on the SF-36 Social functioning (34%) and Role emotional (36%). In lipedema, the ceiling rates were moderate on the SF-36 Role physical, Social functioning, and Role emotional scales. A high ceiling was observed on FLQA-lk Treatment (41%), reflecting that patients did not experience treatment of their condition as a burden.

**Table 2:** Score data and comparison lymphedema (*n* = 107) to lipedema (*n* = 96)

Scale	Abbr	Lymphedema				Lipedema				bivariate/unadjusted				adjusted/multivariate			
		mean	sd	fl	ce	mean	sd	fl	ce	SMD	95%	CI	p	SMD	95%	CI	p
**SF-36**																	
Physical functioning	PF	**70.9**	21.2	–	4	**65.4**	23.3	–	2	**0.25**	−0.03	0.52	0.075	**0.33**	0.05	0.60	0.021
Role physical	RP	**66.3**	24.7	–	18	**60.7**	26.8	2	10	**0.22**	−0.06	0.49	0.116	**0.18**	−0.10	0.46	0.206
Bodily pain	BP	**68.1**	26.7	–	28	**42.4**	23.9	6	4	**1.01**	0.72	1.30	0.000	**0.83**	0.56	1.11	0.000
General health	GH	**60.8**	20.1	–	2	**54.0**	18.8	–	1	**0.35**	0.07	0.62	0.011	**0.30**	0.02	0.58	0.033
Vitality	VT	**54.4**	19.1	–	–	**44.1**	19.5	1	–	**0.53**	0.25	0.81	0.000	**0.39**	0.12	0.67	0.006
Social functioning	SF	**72.1**	27.0	2	34	**64.8**	26.2	2	20	**0.28**	0.00	0.55	0.045	**0.26**	−0.02	0.53	0.071
Role emotional	RE	**72.6**	27.4	1	36	**70.4**	27.2	2	27	**0.08**	−0.19	0.36	0.564	**0.08**	−0.20	0.36	0.580
Mental health	MH	**68.3**	20.2	–	4	**61.5**	19.3	–	1	**0.34**	0.07	0.62	0.012	**0.25**	−0.02	0.53	0.072
PCS	PCS	**46.1**	8.9	–	–	**41.0**	9.5	–	–	**0.55**	0.27	0.83	0.000	**0.53**	0.25	0.80	0.000
MCS	MCS	**45.5**	12.1	–	–	**43.0**	12.2	–	–	**0.20**	−0.07	0.48	0.142	**0.14**	−0.13	0.42	0.307
**FLQA-lk**																	
Physical complaints	Phys	**65.2**	20.9	–	3	**44.9**	17.4	–	–	**1.05**	0.75	1.34	0.000	**0.93**	0.65	1.21	0.000
Everyday life	Ever	**64.3**	24.2	–	7	**60.4**	23.0	–	–	**0.16**	−0.12	0.44	0.249	**0.04**	−0.24	0.32	0.780
Social life	Soci	**76.1**	23.7	–	15	**65.7**	24.4	–	8	**0.43**	0.15	0.71	0.002	**0.30**	0.02	0.58	0.035
Emotional well-being	Emot	**63.4**	21.2	1	2	**52.5**	21.6	1	2	**0.51**	0.23	0.79	0.000	**0.36**	0.09	0.64	0.010
Treatment	Trea	**60.5**	20.0	–	25	**53.0**	19.9	2	41	**0.38**	0.10	0.65	0.006	**0.01**	−0.26	0.29	0.921
Health state (general)	Heal	**77.7**	21.8	–	1	**74.4**	29.5	2	–	**0.13**	−0.15	0.40	0.369	**0.35**	0.07	0.63	0.014
Total score	FLQ	**67.9**	17.7	–	–	**58.5**	17.0	–	–	**0.54**	0.26	0.82	0.000	**0.41**	0.13	0.68	0.004
**KOS-ADL**																	
Symptoms	Sym	**71.9**	21.6	–	6	**62.1**	24.3	1	2	**0.42**	0.15	0.70	0.002	**0.38**	0.10	0.66	0.008
Function	Fun	**73.9**	19.1	–	8	**63.7**	23.7	–	8	**0.48**	0.20	0.75	0.000	**0.47**	0.19	0.74	0.001
Function change	Cha	**70.5**	20.7	1	4	**65.3**	23.8	2	3	**0.23**	−0.05	0.52	0.102	**0.24**	−0.04	0.52	0.087
Total score	KOS	**73.0**	19.2	–	3	**63.0**	22.7	–	–	**0.48**	0.20	0.75	0.000	**0.45**	0.17	0.73	0.002
**SCL-90R**																	
Interpersonal sensitivity	Int	**84.5**	16.4	–	10	**73.2**	21.3	–	3	**0.60**	0.32	0.88	0.000	**0.32**	0.04	0.59	0.026
Obsessive/compulsive	Obs	**84.2**	17.3	–	14	**81.4**	15.6	–	5	**0.17**	−0.11	0.45	0.224	**0.03**	−0.25	0.31	0.820
**6 min walking test**	6 MW	**463.7**	114.0	–	–	**426.1**	141.2	–	–	**0.30**	−0.02	0.62	0.064	**0.28**	−0.05	0.60	0.096

Legend: Scaling of all scores: 0 = worst (maximal symptoms/disability), 100 = best (no symptoms/full function), exception: 6 MWT (meters), *Abbr*: Abbreviation (for use in the appendices), *sd* Standard deviation, *fl* Floor (%), ce = ceiling (%), *SMD* Standardized mean difference between the scores of lymphedema and lipedema, 95% *CI* 95% confidence interval of the SMD, *p* Type I error of the test that the SMD ≠ 0 (2-tailed, independent samples), *SF-36* Short Form 36, *PCS* Physical component summary, *MCS* Mental component summary, *FLQA-lk* Freiburger Lebensqualitäts Assessment-Lympherkrankungen Kurzversion (Freiburg Quality of life Assessment Lymph Disorders, Short Version), *KOS-ADL* Knee Outcome Survey – Activities of Daily Living, *SCL-90R* Symptom Checklist 90 (items) – revised, *6 MWT* 6 min walking test

The mean scores for lymphedema were in the upper half of the possible scale range (0 = worst–100 = best) varying between 54.4 (SF-36 Vitality) and 84.5 (SCL-90R Interpersonal sensitivity); mean scores for lipedema ranged between 42.4 (SF-36 Bodily pain) and 81.4 (SCL-90R Obsessive/compulsive).

### Ability of the scales to specify between lymphedema and lipedema

The outcome of lymphedema was consistently better on all scales compared to lipedema (Table 2), with the differential most marked on the FLQA-lk Physical complaints scale (unadjusted SMD = 1.05, adjusted SMD = 0.93) and the SF-36 Bodily pain scale (unadjusted SMD = 1.01, adjusted SMD = 0.83). It should be remembered that SMDs≥0.30 are statistically significant on the basis of our sample sizes. This level of difference between the outcome of lymphedema and lipedema was observed on SF-36 Physical functioning (unadjusted SMD = 0.25 – not significant, adjusted SMD = 0.33), SF-36 General health (0.35, 0.30), SF-36 Vitality (0.53, 0.39), SF-36 Mental health (0.34, 0.25 – not significant), SF-36 PCS (0.55, 0.53), FLQA-lk Social life (0.43, 0.30), FLQA-lk Emotional well-being (0.51, 0.36), FLQA-lk Health state (0.38, 0.35), FLQA-lk Total score (0.54, 0.41), KOS-ADL Symptoms (0.42, 0.38), KOS-ADL Function (0.48, 0.47), KOS-ADL Total (0.48, 0.45), and SCL-90R Interpersonal sensitivity (0.60, 0.32). The SMDs of the 6 MWT were also above 0.30 (unadjusted SMD = 0.31, adjusted SMD = 0.44) but did not reach statistical significance, owing to the low inpatient sample sizes (*n* = 89 lymphedema, *n* = 64 lipedema).

### Bivariate correlations

Appendices 1 (lymphedema) and 2 (lipedema) present the bivariate correlations. The abbreviations for the scores can be found in Table 2. Moderate (0.70–0.79) or high (≥0.80) correlations between scores of the different instruments were observed for the following scales.

The bivariate correlations for lymphedema were as follows: SF-36 Physical functioning correlated with FLQA-lk Everyday life, KOS-ADL Symptoms, Function and Total score. SF-36 Role Physical correlated with FLQA-lk Everyday life and Total score. SF-36 Bodily pain correlated with FLQA-lk Physical complaints. SF-36 Vitality, SF-36 Role emotional, SF-36 Mental health (highest correlation with FLQA-lk Emotional well-being: 0.79), and SF-36 MCS correlated with FLQA-lk Emotional well-being and Total score. SF-36 Social functioning correlated with FLQA-lk Total score. SF-36 PCS correlated with FLQA-lk, Physical complaints and Everyday life, KOS-ADL Function and Total score. The total scores of the FLQA-lk and the KOS-ADL correlated with each other.

In lipedema most corresponding bivariate correlations were consistently lower than in lymphedema. Across all instruments, the physical scales showed greater divergence from the psychosocial scales than in lymphedema. For example, the correlation of the SF-36 Physical functioning with the SF-36 MCS was 0.42 in lymphedema and 0.05 in lipedema. Between instruments, moderate to high correlations were found between the SF-36-Physical functioning and the KOS-ADL Function and Total score; between both the SF-36 Role physical and PCS and the FLQA-lk Everyday life, the KOS-ADL Symptoms, Function and Total score; and between the SF-36 Bodily pain and the FLQA-lk Total score. In lipedema, unlike lymphedema, the 6 MWT correlated well with the SF-36 Physical functioning, the SF-36 PCS (highest correlation 0.80), the KOS-ADL Symptoms and the KOS-ADL Total score. In lipedema all psychosocial scales showed low correlations of < 0.70.

### Factor analysis

Explorative factor analysis extracted two major health dimensions, physical health and mental health, for both conditions by means of the MAP test and the parallel analysis (Table 3). Both solutions fitted well, explaining total variances of 67.1 and 61.0% respectively. In both conditions, physical health was by far the more important factor.

**Table 3:** Factor solutions reflecting the main construct domains

	Lymphedema	(*n* = 107)	Lipedema	(*n* = 96)
	Physical health	Mental health	Physical health	Mental health
**SF-36**				
**Physical functioning**	**0.83**	0.28	**0.85**	0.02
**Role physical**	**0.72**	0.44	**0.82**	0.24
**Bodily pain**	**0.74**	0.23	**0.73**	0.32
**General health**	0.62	0.27	0.47	0.43
**Vitality**	0.42	**0.78**	0.26	**0.76**
**Social functioning**	0.53	0.58	0.41	0.66
**Role emotional**	0.48	0.65	0.35	0.61
**Mental health**	0.29	**0.84**	0.10	**0.83**
**FLQA-lk**				
**Physical complaints**	**0.76**	0.35	0.55	0.40
**Everyday life**	**0.76**	0.47	**0.75**	0.44
**Social life**	0.43	**0.71**	0.40	**0.73**
**Emotional well-being**	0.46	**0.77**	0.34	**0.81**
**Treatment**	0.34	0.25	0.33	0.31
**Health state**	0.67	0.49	0.64	0.39
**KOS-ADL**				
**Symptoms**	**0.79**	0.22	**0.85**	0.12
**Function**	**0.85**	0.15	**0.84**	0.07
**Function change**	**0.79**	0.14	0.69	0.21
**SCL-90R**				
**Interpersonal sensitivity**	0.10	**0.86**	−0.06	**0.80**
**Obsessive/compulsive**	0.11	**0.84**	0.14	**0.76**
Explained variance (%)	56.8	10.3	47.7	13.3
Explained variance total (%)		67.1		61.0

Legend: see Table 2. Bold are factor loads≥0.70. Excluded are all total scores

In lymphedema, the SF-36 Physical functioning, Role physical and Bodily pain and the FLQA-lk Physical complaints and Everyday life showed high factor loads on physical health (≥0.70) as did all three KOS-ADL scores (56.8% explained variance). Regarding the mental health factor, the high factor loads were on the SF-36 Vitality and Mental health, the FLQA-lk Social Life and Emotional well-being, and both SCL-90R scales (10.3% explained variance).

In lipedema the factor solutions in mental health were very similar (13.3% explained variance) but were slightly different in physical health (47.7% explained variance). The FLQA-lk Physical complaints loaded much lower.

Overall, the greatest convergence (within the factors) occurred between the SF-36 and the KOS-ADL on physical health and between the SF-36 and the SCL-90R on mental health. The greatest divergence (between the factors) emerged between the KOS-ADL and the SCL-90R. Consistent with the bivariate analysis, the divergence between physical and mental/psychosocial dimensions was greater in lipedema than in lymphedema: for example, the loading of SF-36 Physical functioning on mental health was 0.28 in lymphedema but 0.02 in lipedema.

## Discussion

To our knowledge this is the first study to examine and to compare a comprehensive set of generic and condition-specific (edema and leg) outcome measurement scales and to analyze their cross-sectional validity within specific construct dimensions in lymphedema and lipedema. Our detailed findings on the validity of the various scales may help in the process of matching measurement instruments to the aims and scope of the outcome examination.

Overall, the generic SF-36 scale showed high content and construct convergence with the condition-specific tools in both physical and mental health dimensions (convergent validity) and good ability to differentiate between lymphedema and lipedema (discriminant validity). As mentioned earlier, since the validity of the SF-36 is best proven, it serves also as criterion for validity for the other instruments. As expected, the ability to specify between lymphedema and lipedema was greatest in pain.

Since, by definition, pain characterizes lipedema but not lymphedema, the discriminant validity of the FLQA-lk Physical complaints (which includes pain) and the SF-36 Bodily pain represents a “known-groups” validity, which also has the characteristic of criterion validity [13]. The “gold standard” criterion is the diagnosis lymphedema versus lipedema, characterized by the presence or absence of pain. The ability of the two above-mentioned scales to specify and to discriminate between the two conditions remained stable even after multivariate adjustment for confounders. Construct divergence emerged between the physical and the mental health scales, especially so for the SF-36, the KOS-ADL and the SCL-90R, and to a slightly lesser degree for the FLQA-lk (see factor analysis).

The FLQA-lk, designed specifically for lymphedema, aligned well with the other tools in measuring that condition. When measuring outcome in lipedema, however, it represented a slightly different construct compared to the other tools (see factor analysis). The FLQA-lk’s ability to specify/differentiate between the two conditions was high on four of the six scales and on the Total score. In both lymphedema and lipedema, the KOS-ADL was confirmed as a pure knee-specific tool and the SCL-90R as a pure mental health measure. The KOS-ADL was the most powerful instrument for the specification of lipedema from lymphedema, especially on function, whereas only the Interpersonal sensitivity scale of the SCL-90R differentiated satisfactorily between the two diagnoses. There was a much higher construct overlap between the 6 MWT and the function scales of the SF-36 and the KOS-ADL Physical complaints and Total score in lipedema than in lymphedema. The 6 MWT’s potential to differentiate between the two conditions was moderate but as a result of the smaller inpatient sample sizes, the SMDs did not reach statistical significance.

The comparable study by Van den Pas, which was confined to the correlations between the LYMQOL and the two SF-36 component summary scales (PCS and MCS) in lymphedema of the leg (*n* = 67), found correlations similar to our own for physical health [8]: The correlations with the PCS established by Van den Pas were: symptoms 0.66 (bivariate coefficients, our data: 0.62 to 0.70), function 0.73 (0.71), mood/emotions 0.62 (0.40), and overall quality of life 0.50 (0.58–0.63). The correlations with the MCS, however, diverged from our findings: symptoms 0.28 (our data: 0.41 to 0.50), function 0.33 (0.36 to 0.59), mood/emotions 0.42 (0.82), and overall quality of life 0.31 (0.36 to 0.73).

Our study has several strengths. It is the first study to test both comprehensive and specific outcome measurement using five standardized, validated instruments in lymphedema and lipedema. The results provide a basis for recommendations on the establishment of assessment sets. In comparison to other studies, our sample sizes were large enough to ensure that minimally clinically important differences reached statistical significance and to fulfil the requirements for valid factor analysis. Beyond bivariate correlation analysis, multivariate factor analysis explored the most important dimensions of convergent and divergent constructs and provided dimension reduction to facilitate interpretation. Discriminant validity was examined by adjusted differences between the two diseases, giving results that are independent from unequally distributed confounding characteristics and more readily generalizable.

The limitations of our study were its cross-sectional design, which excludes the dimension of outcome changes over time, and the fact that the 6 MWT was only available for the inpatient sub-sample. Our findings on cross-sectional validity need to be completed by validity data from longitudinal studies and especially by analysis of the sensitivity to change (responsiveness).

## Conclusions

This study provides unique elaborated data on the validity of the instruments used for the comprehensive and specific outcome measurement of lipedema and lymphedema. We would recommend that a core assessment set should include the following nine scales: SF-36 Bodily pain, SF-36 Vitality, FLQA-lk Physical complaints, FLQA-lk Social life, FLQA-lk Emotional well-being, FLQA-lk Health state, KOS-ADL Symptoms, KOS-ADL Function, and SCL-90R Interpersonal sensitivity. Further measurement needs can be met by complementing this basic scale set with additional constructs, for example the SF-36 Role physical (dimension of role performance), the FLQA-lk Treatment (for distress due to treatment), the SCL-90R Obsessive/compulsive, or the 6 MWT (examiner-based function). For an exhaustive assessment, including the total scores, the full set comprising all five complete instruments can be used.

## Data Availability

All data and material are free available. Please contact the corresponding author for data requests.

## Appendix 1

**Table 4:** Bivariate correlations of scores in lymphedema (*n* = 107)

	SF-36										FLQA-lk							KOS-ADL				SCL-90R	6 MW	
	PF	RP	BP	GH	VT	SF	RE	MH	PCS	MCS	Phys	Ever	Soci	Emot	Trea	Heal	FLQ	Sym	Fun	Cha	KOS	Int	Obs	6 MW
PF	1.00	**0.77**	0.64	0.53	0.54	0.58	0.58	0.46	**0.84**	0.42	0.66	**0.75**	0.52	0.54	0.19	0.65	0.69	**0.70**	**0.78**	0.65	**0.78**	0.40	0.37	0.55
RP		1.00	0.61	0.55	0.69	0.61	**0.73**	0.55	**0.76**	0.58	0.66	**0.75**	0.61	0.64	0.33	0.62	**0.75**	0.57	0.64	0.60	0.64	0.42	0.43	0.47
BP			1.00	0.43	0.49	0.51	0.42	0.40	**0.80**	0.34	**0.75**	0.66	0.51	0.47	0.24	0.58	0.66	0.59	0.58	0.53	0.61	0.31	0.33	0.28
GH				1.00	0.49	0.46	0.42	0.35	0.67	0.37	0.53	0.60	0.43	0.53	0.26	0.61	0.61	0.47	0.53	0.43	0.53	0.31	0.29	0.12
VT					1.00	0.66	0.69	**0.82**	0.42	**0.84**	0.63	0.68	0.66	**0.81**	0.30	0.67	**0.78**	0.43	0.45	0.43	0.46	0.62	0.60	0.34
SF						1.00	0.63	0.64	0.47	**0.76**	0.58	0.66	0.69	0.67	0.44	0.63	**0.76**	0.49	0.45	0.54	0.49	0.44	0.47	0.34
RE							1.00	0.68	0.35	**0.85**	0.54	0.63	0.62	**0.72**	0.28	0.63	**0.71**	0.50	0.51	0.49	0.53	0.52	0.55	0.38
MH								1.00	0.21	**0.91**	0.51	0.57	0.64	**0.79**	0.24	0.62	**0.70**	0.46	0.39	0.40	0.44	0.69	0.66	0.19
PCS									1.00	0.16	**0.70**	**0.71**	0.44	0.40	0.24	0.58	0.63	0.62	**0.71**	0.60	**0.70**	0.22	0.21	0.40
MCS										1.00	0.50	0.59	0.68	**0.82**	0.32	0.63	**0.73**	0.41	0.36	0.43	0.40	0.64	0.65	0.26
Phys											1.00	**0.74**	0.57	0.63	0.34	0.64	**0.81**	0.63	0.65	0.59	0.68	0.38	0.38	0.42
Ever												1.00	**0.73**	**0.70**	0.41	**0.70**	**0.89**	0.66	0.65	0.65	0.69	0.47	0.46	0.34
Soci													1.00	**0.74**	0.50	0.56	**0.86**	0.46	0.42	0.45	0.46	0.64	0.57	0.17
Emot														1.00	0.36	**0.77**	**0.86**	0.52	0.48	0.48	0.53	0.65	0.59	0.24
Trea															1.00	0.28	0.60	0.25	0.28	0.31	0.28	0.13	0.19	−0.12
Heal																1.00	**0.81**	0.61	0.62	0.58	0.65	0.44	0.45	0.40
FLQ																	1.00	0.65	0.64	0.66	**0.78**	0.57	0.55	0.29
Sym																		1.00	**0.81**	0.62	**0.94**	0.39	0.35	0.44
Fun																			1.00	0.63	**0.96**	0.30	0.31	0.45
Cha																				1.00	0.68	0.20	0.19	0.60
KOS																					1.00	0.36	0.35	0.47
Int																						1.00	**0.83**	0.15
Obs																							1.00	0.15
6 MW																								1.00

Legend: Abbreviations of the scales: see Table 2, 2^nd^ column. Correlations: Pearson’s product moment. Bold are correlations≥0.70

## Appendix 2

**Table 5:** Bivariate correlations of scores in lipedema (*n* = 96)

	SF-36										FLQA-lk							KOS-ADL				SCL-90R	6 MW	
	PF	RP	BP	GH	VT	SF	RE	MH	PCS	MCS	Phys	Ever	Soci	Emot	Trea	Heal	FLQ	Sym	Fun	Cha	KOS	Int	Obs	6 MW
PF	1.00	0.67	0.55	0.33	0.26	0.45	0.34	0.08	**0.85**	0.05	0.45	0.67	0.39	0.29	0.14	0.48	0.52	0.66	**0.74**	0.52	**0.74**	0.00	0.20	**0.75**
RP		1.00	0.65	0.42	0.38	0.51	0.57	0.26	**0.79**	0.28	0.47	**0.72**	0.54	0.40	0.30	0.54	0.65	**0.70**	**0.70**	0.56	**0.74**	0.16	0.34	0.53
BP			1.00	0.38	0.45	0.51	0.37	0.38	**0.74**	0.28	0.64	0.63	0.43	0.54	0.45	0.61	**0.72**	0.63	0.54	0.55	0.61	0.20	0.29	0.39
GH				1.00	0.46	0.41	0.41	0.39	0.48	0.40	0.42	0.54	0.49	0.49	0.26	0.47	0.58	0.42	0.34	0.49	0.39	0.23	0.31	0.41
VT					1.00	0.63	0.47	0.69	0.25	**0.73**	0.60	0.50	0.56	0.68	0.33	0.47	0.68	0.24	0.25	0.36	0.25	0.43	0.52	−0.23
SF						1.00	0.59	0.61	0.36	**0.72**	0.38	0.56	0.67	0.60	0.43	0.44	0.69	0.40	0.40	0.34	0.42	0.41	0.46	−0.12
RE							1.00	0.60	0.19	**0.79**	0.27	0.52	0.59	0.51	0.19	0.34	0.53	0.40	0.38	0.33	0.41	0.40	0.48	−0.11
MH								1.00	− 0.04	**0.91**	0.37	0.36	0.55	0.69	0.30	0.38	0.59	0.24	0.17	0.28	0.21	0.53	0.53	−0.27
PCS									1.00	−0.10	0.55	**0.70**	0.36	0.29	0.28	0.56	0.59	**0.71**	**0.72**	0.59	**0.75**	−0.05	0.15	**0.80**
MCS										1.00	0.30	0.37	0.61	0.66	0.28	0.32	0.57	0.18	0.13	0.21	0.16	0.56	0.56	−0.41
Phys											1.00	0.55	0.39	0.63	0.21	0.54	0.69	0.42	0.46	0.43	0.46	0.22	0.37	0.28
Ever												1.00	**0.70**	0.64	0.36	0.66	**0.85**	0.62	0.65	0.59	0.67	0.35	0.48	0.50
Soci													1.00	**0.70**	0.26	0.53	**0.79**	0.42	0.40	0.45	0.43	0.61	0.61	−0.06
Emot														1.00	0.37	0.62	**0.86**	0.34	0.30	0.42	0.34	0.58	0.61	−0.05
Trea															1.00	0.25	0.59	0.38	0.20	0.35	0.29	0.18	0.18	0.07
Heal																1.00	0.77	0.51	0.46	0.67	0.51	0.20	0.36	0.56
FLQ																	1.00	0.59	0.53	0.63	0.59	0.48	0.58	0.28
Sym																		1.00	**0.80**	0.62	**0.93**	0.13	0.32	**0.70**
Fun																			1.00	0.50	**0.96**	0.11	0.28	0.68
Cha																				1.00	0.58	0.18	0.21	0.69
KOS																					1.00	0.12	0.32	**0.71**
Int																						1.00	**0.75**	−0.44
Obs																							1.00	−0.18
6 MW																								1.00

Legend: Abbreviations of the scales: see Table 2, 2^nd^ column. Correlations: Pearson’s product moment. Bold are correlations≥0.70

## Abbreviations

6 MWT: Six-minute Walking Test
BMI: Body Mass Index
CEAP: Clinical, Etiology, Anatomic location, Pathophysiology
EK AG: Ethics (K) committee of the canton AarGau
FLQA-lk: Freiburg Quality of Life Assessment for lymphatic disorders, Short Version
KOS-ADL: Knee Outcome Survey Activities of Daily Living Scale
LYMQOL: Lymphedema Quality-of-Life Questionnaire
M: Meters
MAP: Velicer’s Minimum Average Partial test
MCID: Minimal Clinically Important Difference
MCS: Mental Component Summary (of the SF-36)
N: Number (of patients)
PCS: Physical Component Summary (of the SF-36)
SCL-90R: Symptom CheckList 90 – Revised
SF-36: Short Form 36
SMD: Standardized Mean Difference
SW: Stephan Wagner (co-author)
